# Temporal variation in nutrient requirements of tea (*Camellia sinensis*) in China based on QUEFTS analysis

**DOI:** 10.1038/s41598-020-57809-x

**Published:** 2020-02-04

**Authors:** Sheng Tang, Yanling Liu, Nan Zheng, Yu Li, Qingxu Ma, Han Xiao, Xuan Zhou, Xinpeng Xu, Taiming Jiang, Ping He, Lianghuan Wu

**Affiliations:** 1 Zhejiang Provincial Key Laboratory of Agricultural Resources and Environment, College of Environmental and Resource Sciences, Zhejiang University, Hangzhou, 310058 China; 2 State Key Laboratory of Nutrition Resources Integrated Utilization, Kingenta Ecological Engineering Group Co. Ltd., Linyi, 276000 Shandong China; 3 Institute of Soil and Fertilizer, Guizhou Academy of Agricultural Sciences, Guiyang, 550006 China; 4 Institute of Soil and Fertilizer, Hunan Academy of Agricultural Sciences, Changsha, 410125 China; 5 Ministry of Agriculture Key Laboratory of Crop Nutrition and Fertilization, Institute of Agricultural Resources and Regional Planning, Chinese Academy of Agricultural Sciences (CAAS), Beijing, 100081 China; 6 Institute of Tea research, Guizhou Academy of Agricultural Sciences, Guiyang, 550006 China

**Keywords:** Physiology, Plant sciences, Plant physiology

## Abstract

Fertilisation datasets collected from field experiments (*n* = 21) in tea-producing areas from 2016 to 2018 were used to build a quantitative evaluation of the fertility of tropical soils (QUEFTS) model to estimate nutrient uptake of tea plants, and to investigate relationships between tea yield and nutrient accumulation. The production of 1000 kg spring tea (based on one bud with two young expanding leaves) required 12.2 kg nitrogen (N), 1.2 kg phosphorus (P), and 3.9 kg potassium (K), and the corresponding internal efficiencies (IEs) for N, P, and K were 82.0, 833.3, and 256.4 kg kg^−1^. To produce 1000 kg summer tea, 9.1 kg N, 0.8 kg P, and 3.1 kg K were required, and the corresponding IEs for N, P, and K were 109.9, 1250.0, and 322.6 kg kg^−1^. For autumn tea, 8.8 kg N, 1.0 kg P, and 3.2 kg K were required to produce 1000 kg tea, and the corresponding IEs for N, P, and K were 113.6, 1000.0, and 312.5 kg kg^−1^. Field validation experiments performed in 2019 suggested that the QUEFTS model can appropriately estimate nutrient uptake of tea plants at a certain yield and contribute to developing a fertiliser recommendation strategy for tea production.

## Introduction

Tea (*Camellia sinensis*) is the most consumed manufactured drink worldwide, and the plant is widely grown in tropical and subtropical areas, especially in Asian, African, and Latin American countries. It is a perennial crop whose leaves are harvested, and which grows effectively in acidic soil with an optimum pH of 4.0–5.5^1^. In 2013, global tea production reached over 5 million tons, and its consumption continues to increase, particularly in China and India^2^. The use of nitrogen (N) fertiliser has been shown to improve tea yield and quality, especially for the synthesis of amino acids critical for producing high-quality green tea^3,4^, and the metabolism of minerals and metabolite products is influenced by phosphate (P) uptake^5^. Potassium (K) plays vital roles in tea yield and the synthesis of amino acids and caffeine^6^, and application of K fertiliser can significantly increase water-extractable dry matter^7^. Tea trees require a high N supply, with the current fertilisation rate ranging from 450 to 1200 kg N ha^−1^ year^−1^ ^8–10^, much more than that used in other artificial ecosystems. Recent research suggested that the excessive application of chemical fertiliser in tea gardens in China is a major problem. More than 30% of the tea garden area is over-sprayed, and more than 50% of the tea gardens have high P and K nutrient input, with an unreasonable proportion of nutrients^11^. Concurrently, massive amounts of N not taken up by plants can be lost to adjacent systems, causing serious environmental problems in tea-planted areas^5,7,12,13^. Moreover, an unbalanced soil nutrient status can limit plant growth and formation of biochemical ingredients in tea^14^. Therefore, an optimum fertiliser recommendation for tea cultivation should focus on both ensuring high crop yield and reducing environmental risk, so as to maintain sustainable agriculture.

Estimating nutrient requirements of tea plants is essential for rational fertilisation^15^, but it is difficult to determine nutrient requirements based on limited experimental data. Because the eco-environment of tea plants varies substantially in terms of soil type, climate condition, nutrient supply, and tea varieties, interactions between N, P, and K in tea plantations can be difficult to calculate^7^. Recently, a quantitative evaluation of the fertility of tropical soils (QUEFTS) model has been successfully used to estimate the nutrient requirements of various crops, and to provide recommendations for fertiliser application during their cultivation^16,17^. This approach has been applied to maize^18–20^, rice^15,21,22^, wheat^20,23^, and radish^24^. The QUEFTS model avoids deviation when minimal data are used to obtain nutrient uptake information to guide fertiliser application for crops^19^. The model also considers interactions between uptake rates and crop requirements of N, P, and K^25^. Therefore, it provides a practical tool for site-specific nutrient management for major crops^18,26^. Constants (a) and (d) respectively represent maximum accumulation (equivalent to the minimum internal nutrient efficiencies, or IEs) and maximum dilution (equivalent to maximum IEs) of N, P, and K as model parameters to evaluate balanced nutrient requirements according to the model. However, the QUEFTS model has not yet been applied to the cultivation of perennial crops such as tea.

In China, tea production is classified as spring (late May and earlier), summer (early June to early July), or autumn (mid-July and later) based on its date of harvest^27^. The chemical composition and taste quality of tea fluctuate seasonally^28^. Generally, spring tea has a higher amino acid content and moderate levels of catechins, yielding a heavy, mellow, and brisk flavour^29^. However, an equivalent-quantification of tea tastes has shown that the bitterness and astringency are significantly elevated in summer and autumn tea, while its umami flavour declines, resulting in a sharp drop in price^28^. After spring tea leaves are harvested, summer and autumn tea leaves remain; because these tend to have a bitter flavour, they are used to make lower-value products and command a much lower market price. For example, in Sichuan, the spring tea yield in 2015 was 13.41 million tons, with a value of 11.9 billion RMB, while the summer and autumn tea yield was 11.43 million tons, with a value of 3.8 billion RMB^30^. Improving the flavour and yield of summer and autumn tea crops could increase the annual profitability of tea production^31^. As the chemical composition of tea leaves is inextricably linked to nutrient absorption, timely application of top-dressing fertilisers may improve tea quality and yield^32^. However, there have been few studies dealing with nutrient uptakes of summer and autumn tea. We conducted field experiments with different climates, soil types, and varieties of tea plants over four years (2016–2019). The data collected from the experiments conducted in the first three years (2016–2018) were used to construct the QUEFTS model, and the data from the final year (2019) were used to validate the QUEFTS model. We assessed these data using a QUEFTS model to (1) explore temporal variation in fertiliser requirement among different harvest seasons of tea; (2) evaluate nutrient uptakes simulated by the QUEFTS model; and (3) recommend optimum fertilisation strategies based on nutrient uptake and yield over all three tea harvest seasons.

## Results

### Yield and nutrient uptake

In this study, ‘all tea’ represented the total annual tea including spring, summer, and autumn tea. The average yield (based on one bud with two young expanding leaves, adjusted to 75% moisture content) of spring, summer, autumn, and all tea from 2016 to 2018 was 1787 kg ha^−1^, 2084 kg ha^−1^, 1583 kg ha^−1^, and 8164 kg ha^−1^, respectively (Supplementary Fig. S1). Summer tea yield was significantly higher than spring and autumn tea yield (*p* < 0.05), but there were no significant differences between spring and autumn tea yield. The average rates of N, P_2_O_5_, and K_2_O application were 321 kg ha^−1^, 151 kg ha^−1^, and 137 kg ha^−1^, respectively.

The average IEs (kg yield per kg nutrient uptake) of N, P, and K for spring tea were 75.7 kg kg^−1^, 733.2 kg kg^−1^, and 239.0 kg kg^−1^, respectively (Supplementary Table S1), and the corresponding RIEs (reciprocal internal efficiency, nutrient uptake requirement per ton of yield) were 13.6 kg t^−1^, 1.4 kg t^−1^, and 4.4 kg t^−1^ with an N:P:K ratio of 9.4:1:3.0. For summer tea, the average IEs of N, P, and K were 101.7 kg kg^−1^, 1134.1 kg kg^−1^, and 264.8 kg kg^−1^, respectively, with RIEs of 10.0 kg t^−1^, 0.9 kg t^−1^, and 4.0 kg t^−1^ with a ratio of 10.9:1:4.3. For autumn tea, the average IEs of N, P, and K were 106.0 kg kg^−1^, 1063.9 kg kg^−1^, and 274.8 kg kg^−1^, respectively (Supplementary Table S1), with RIEs of 9.7 kg t^−1^, 0.99 kg t^−1^, and 3.9 kg t^−1^ with a ratio of 9.8:1:3.9. The IEs of N, P, and K for the three seasons were significantly different (*p* < 0.05). The IEs of N and K were ranked as autumn > summer > spring, while P was ranked as summer > autumn > spring. Correspondingly, the RIEs of N and K in spring tea were significantly higher than those of summer and autumn tea. In addition, there were significant differences between the RIEs of P for spring, summer, and autumn tea (*p* < 0.05), and the RIEs could be ranked as spring > autumn > summer.

### Calculating the parameters for running the QUEFTS models

**Figure 1:**
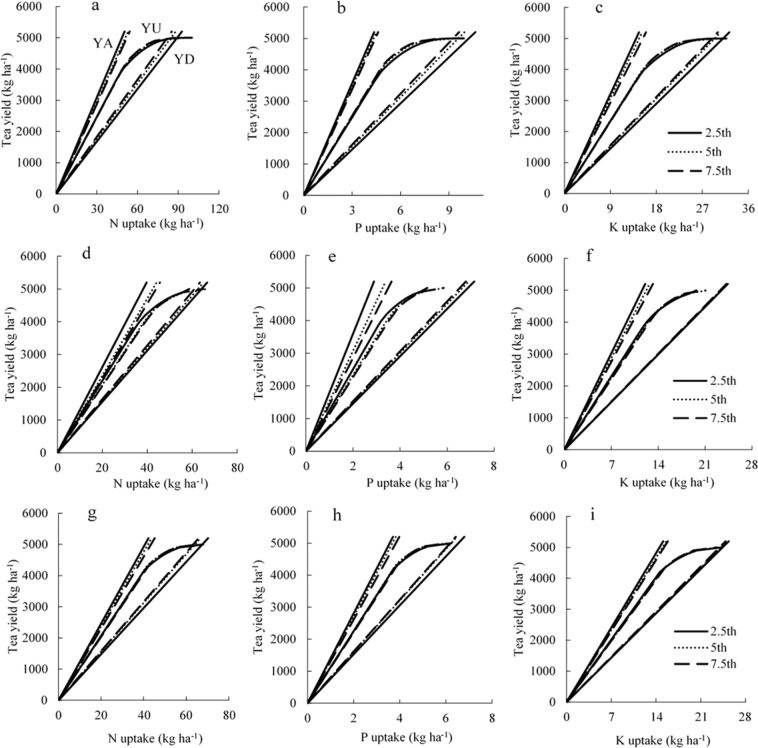
The nutrient requirements of spring (**a**–**c**), summer (**d**–**f**), and autumn (**g**–**i**) tea simulated by QUEFTS models based on Set I, Set II, and Set III, respectively. The yield potential of tea was set at 5000 kg ha^−1^. YD, YA, and YU are the maximum dilution, maximum accumulation, and balanced uptake of N, P, and K in tea, respectively.

In Supplementary Table S2, (a) and (d) were calculated by excluding the upper and lower 2.5 (Set I), 5.0 (Set II), and 7.5 (Set III) percentiles of all IE data of the combined datasets. We estimated the nutrient requirements for spring, summer, autumn, and all tea for a specific targeted tea yield based on Sets I-III (Fig. 1). The nutrient requirements simulated by the three series were similar for spring, summer, autumn, and all tea (Fig. 1, Supplementary Table S2). Set I was selected to run the QUEFTS model to estimate the relationship between tea yield and nutrient requirements, as it contained a larger range of variability than did Sets II and III. The values of (a) in N, P, and K requirements were 56, 489, and 161 kg kg^−1^ for spring tea; 78, 727, and 213 kg kg^−1^ for summer tea; 75, 642, and 184 kg kg^−1^ for autumn tea; and 74, 764, and 205 kg kg^−1^ for all tea, respectively. The values of (d) for N, P, and K were 103, 1193, and 356 kg kg^−1^ for spring tea; 131, 1798, and 431 kg kg^−1^ for summer tea; 141, 1436, and 409 kg kg^−1^ for autumn tea; and 123, 1404, and 344 kg kg^−1^ for all tea.

### Temporal variations in nutrient requirements

**Figure 2:**
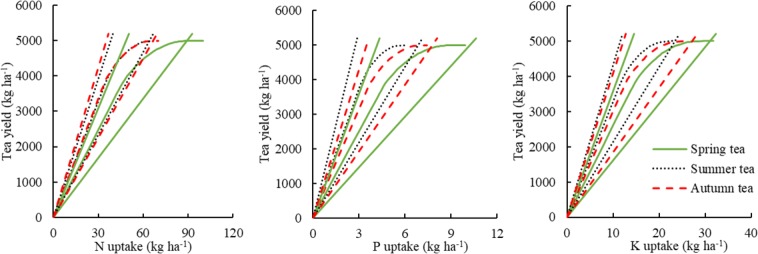
At the tea yield of 5000 kg ha^−1^, the nutrient requirements of spring, summer, and autumn tea estimated by QUEFTS based on Set I.

Based on the above results, Set I was used to estimate the nutrient requirements for spring, summer, and autumn tea at the yield potential (maximum attainable yield) of 5000 kg ha^−1^ (Fig. 2). At this yield potential, there were significant differences in N requirements between spring tea and other teas (*p* < 0.01). In other words, to attain the same targeted tea yield in the range of 0 to 5000 kg ha^−1^, spring tea required more N than did summer or autumn tea. However, no significant differences in N requirement were observed between summer and autumn tea (*p* > 0.05). For P and K uptake, there were significant differences across all seasons (*p* < 0.05). In general, to produce the same tea yield, seasonal P and K requirements could be ranked as summer < autumn < spring. At this yield potential, spring tea required relatively more N, P, and K (Fig. 2). To produce 1000 kg tea, spring tea required 12.2 kg N, 1.2 kg P, and 3.9 kg K in a ratio of 10.1:1:3.2; summer tea required 9.1 kg N, 0.8 kg P, and 3.1 kg K in a ratio of 11.3:1:3.8; and autumn tea required 8.8 kg N, 1.0 kg P, and 3.2 kg K in a ratio of 9.2:1:3.4 (Supplementary Fig. S2).

### Evaluating the relationship between yield and nutrient uptake

**Figure 3:**
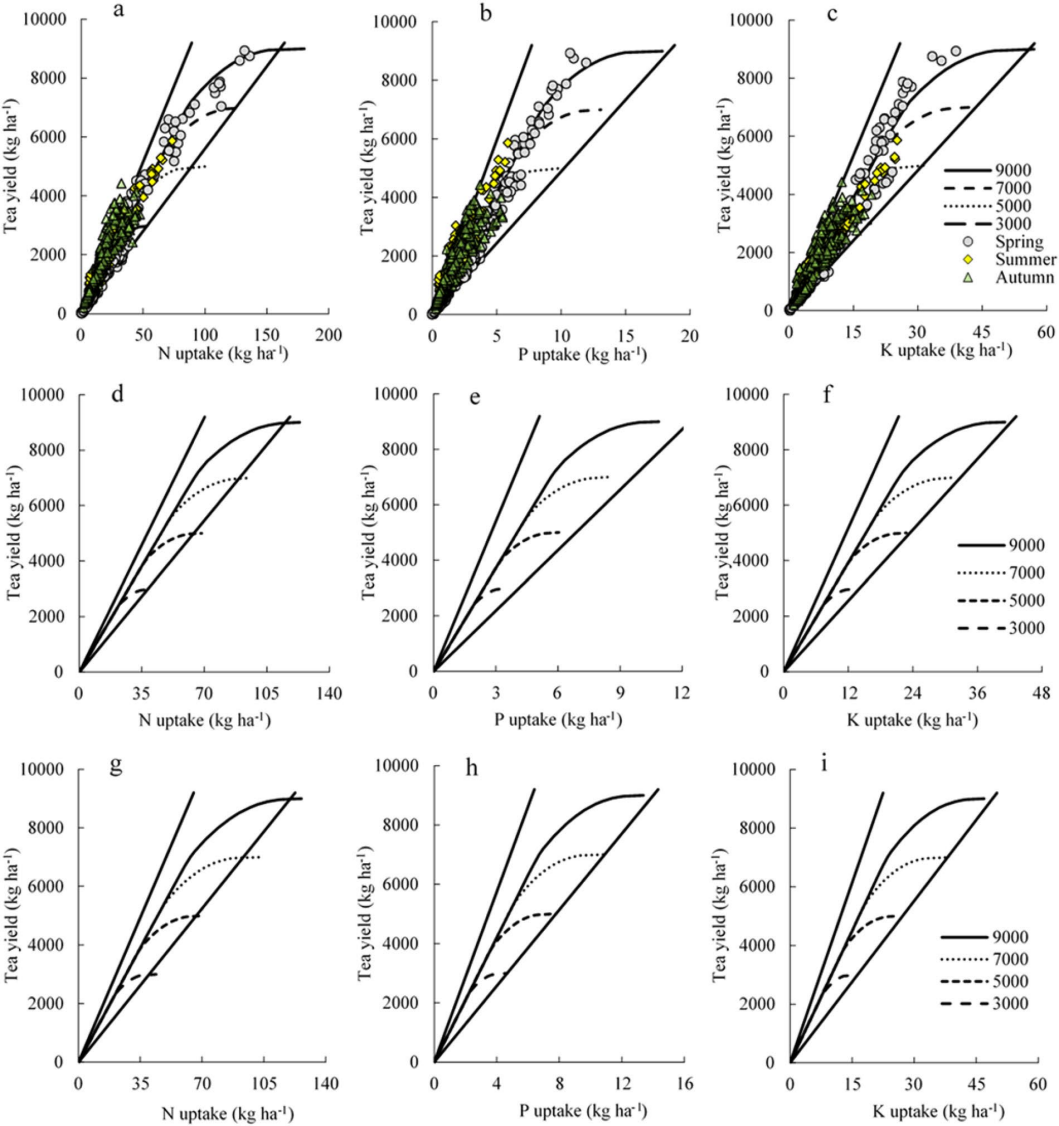
Nutrient requirements of spring (**a**–**c**), summer (**d**–**f**), and autumn (**g**–**i**) tea of different tea yield potentials simulated by QUEFTS model.

**Figure 4:**
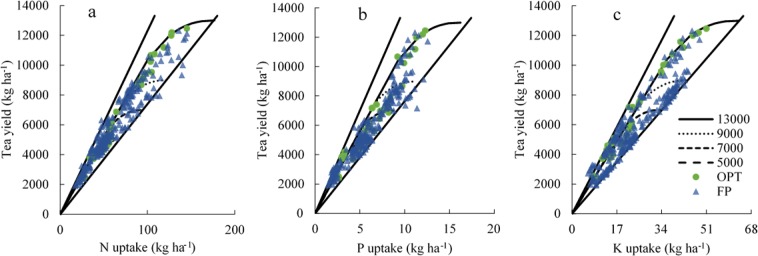
N (**a**), P (**b**), and K (**c**) requirements for all tea with the tea yield potentials set as ranging from 5000 to 13000 kg ha^−1^. The datasets shown were collected from all experiments from 2016 to 2018.

The tea yield potentials were set as ranging from 3000 to 9000 kg ha^−1^ for spring, summer, and autumn tea, based on the yields recorded in field experiments (Fig. 3). The points distributed in Fig. 3 were recorded in all field experiments. N uptake for summer and autumn tea was almost distributed above the N absorption curve of spring tea (Fig. 3a), indicating that summer and autumn tea require less N than does spring tea to achieve the same yield. Most of the observations of P and K uptake for summer and autumn tea were distributed under the uptake curve of spring tea (Fig. 3b,c). Therefore, P and K requirements of summer and autumn tea were less than those of spring tea to achieve the same yield. The yield potential of all tea was set as ranging from 5000 to 13,000 kg ha^−1^ (Fig. 4). Most optimum practice treatment (OPT) practices were distributed around the nutrient uptake curve (Fig. 4). This indicated that the fertiliser application of OPT was rational for tea plant requirements. However, farmers’ practices (FP) approaches to fertilisation were distributed above and below the nutrient uptake curve, indicating that the fertiliser application of FP was not in balance.

### QUEFTS model validation

**Figure 5:**
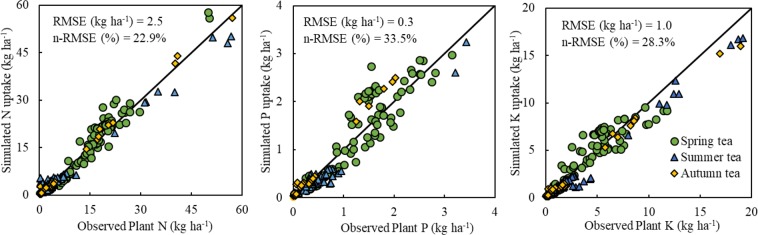
Relationships between the observed and simulated N, P, and K uptake for spring, summer, and autumn tea. The observed nutrient uptake is from tea expert-based fertiliser recommendations in Anhui, Hubei, Hunan, Zhejiang, Fujian, Yunnan, Sichuan, and Guizhou provinces, and the simulated nutrient uptake was derived from the QUEFTS model.

The datasets collected from OPT experiments conducted in 2019 were used to validate the QUEFTS model (Fig. 5). The tea yield and nutrient uptake data from 2019 are listed in Supplementary Table S3. OPT approaches and QUEFTS predictions were distributed around the 1:1 line. The parameters of root mean square error (RMSE) and normalised RMSE (n-RMSE) were used to evaluate the QUEFTS model and the deviation between the observations and simulated data. The RMSE values of N, P, and K were 2.5, 0.3, and 1.0 kg ha^−1^, respectively, and the n-RMSE values of N, P, and K were 22.9%, 33.5%, and 28.3%. The relatively lower values of RMSE and n-RMSE indicated that the data simulated by QUEFTS were similar to the observations. There were no significant differences between observed and simulated data (*p* > 0.05). This indicates that the nutrient uptake of tea plants can be appropriately predicted by QUEFTS at a targeted yield.

## Discussion

In this study, we chose one bud with two young expanding leaves as our harvest standard, which is commonly used among the main tea production regions. In addition to this standard, there are harvest standards such as one bud with three expanding leaves, one bud and so on. Therefore, the QUEFTS model is not able to adapt for all the harvest standards. The yield potential of tea was set at 5000 kg ha^−1^ as an example, as the yield potential has no effect on sensitivity testing^23,33^. The N requirement was about 10 times the P requirement and 2.5 times the K requirement, according to the RIE ratio. Previous studies have found that the RIE ratio of N, P, and K simulated using QUEFTS was 5.08:1:0.97 for wheat, 4.65:1:3.88 for maize, and 4.98:1:5.54 for rice in China^15,19,23^. As a typical leaf-based crop, tea plants require sufficient macronutrients to meet the demands of leaf growth and synthesis of metabolites^34^. Tea also has a comparatively greater N requirement relative to other crops^35,36^.

The RIEs simulated by our QUEFTS models were constant only when the targeted yield was below 60–70% of the yield potential, and increased as the targeted yield exceeded this threshold, as has been found in previous studies^15,19,22,23^. However, the RIEs of N, P, and K for different seasons predicted by QUEFTS differed from those reported in previous studies. The RIEs of N, P, and K estimated by Pan^32^ were 14.3, 0.7, and 4.3 kg t^−1^, respectively; the values for N and K estimated by Pan were similar to the mean measured nutrient uptake of N and K for spring tea, at 13.6 and 4.4, respectively. The differences in RIE could be due to the tea variety cultivated, climate condition, harvest standard, fertiliser application regime, and other factors^37–40^. A previous survey found that the annual N application rate ranged from 0 to 2600 kg ha^−1^ with an average of 553 kg ha^−1^ in typical tea fields of China^41^. For our field experiments, the average N, P_2_O_5_, and K_2_O application rates appeared rational in terms of the data collected from various fertilisation situations including FP, OPT, and nutrient omission plots.

The above results suggest that spring tea has a greater nutrient requirement than do summer or autumn tea (Fig. 2, Supplementary Fig. S2). Correspondingly, the N, P, and K content in spring tea leaves were greater than those in summer and autumn tea leaves, and the RIEs of summer tea were similar to those of autumn tea. The relatively higher nutrient content in new shoots of spring tea, in comparison with those in the shoots of summer and autumn tea, results in greater concentrations of metabolites such as amino acids and polyphenols, which benefit tea quality^2,27^. Spring provides comparably favourable weather, with steadily rising temperature and high humidity, which are suitable for new shoot growth in tea^42,43^. In addition, long-term nutrient accumulation in tea plants from late autumn to early spring contributes to sufficient nutrient storage. The nutrients supplied by basal application of fertilisers as well as top dressing before spring tea harvest also possibly play a role in greater nutrient uptake by tea plants during spring. By contrast, warmer and drier weather in summer and autumn is less favourable for the growth of new shoots^43^, and the nutrients in tea plants decrease after spring tea harvest^27,32^. These factors account for the relatively higher RIEs of spring tea. Moreover, the nutrient uptake of tea fluctuates with seasons, affecting the chemistry and quality of tea leaves^28,43,44^. Wang *et al*.^43^ found that daily average temperature, relative humidity, and precipitation had significant effects on the synthesis of tea polyphenols. The tea polyphenols (TP) to total free amino acids (AA) ratio, which is inversely correlated with the quality of green tea, increases in summer because of higher temperatures^45,46^. Based on meteorological data and statistical data of tea yield and quality, Jin *et al*.^47,48^ found a close relationship between climate and tea yield and quality.

Tea quality is affected by both fertiliser management and regional climate^43^, and the relatively higher TP/AA ratio in summer and autumn tea results in a sharp price decrease^27^. Rational fertilisation to improve the yield and quality of tea could increase farmers’ income. The ratio of RIE of N for spring, summer, and autumn tea was approximately 3:2:2, the same as in the top-dressing application of N fertiliser. P and K removed from tea shoots should be returned by fertiliser application to avoid soil nutrient depletion as well as minimise environmental risk.

Fertilisation evaluation can also be combined with a QUEFTS model^15,23^. Our comparison indicated that nutrient provision was insufficient when practice datasets were distributed above the nutrient uptake curve and close to the upper boundary, and our model predicted a potentially greater tea yield under increased nutrient application. In contrast, if the datasets are distributed below the nutrient uptake curve and close to the lower boundary, it might indicate excessive nutrient provision, with tea yield being limited by other growth factors (Fig. 4). Most of the observations of N and P uptake of FP indicated luxury absorption, suggesting that N and P fertiliser application were excessive in FP of tea planting. In addition, K fertiliser application in FP was excessive in some areas and deficient in others. These observations are consistent with the findings of previous studies^11,14^. Overall, these results suggest that fertiliser application can be recommended based on the nutrient uptake of tea plants.

## Conclusions

In summary, we found significant differences in N, P, and K uptake across spring, summer, and autumn tea (*p* < 0.05), and the nutrient uptake characteristics varied across harvest seasons. In order to attain a targeted yield (based on one bud with two young expanding leaves), the nutrient requirements of spring tea were relatively greater than those in summer or autumn, but there were no significant differences between summer and autumn tea in this regard (*p* > 0.05). The fertilisation practice datasets collected in our study represented a wide range of tea-growing environments in China, with different values of (a) and (d) for spring, summer, and autumn tea. Field validation indicated that the QUEFTS model could be used to estimate N, P, and K uptake of tea plants at a precise targeted yield. Regardless of yield potential, the model predicted a linear increase in tea yield if there is a balanced nutrient uptake until the yield reaches about 60–70% of the yield potential. Thus, fertiliser recommendation for tea using a QUEFTS models could help to avoid excessive fertilisation and reduce its effect on the environment and improve tea yield.

## Materials and Methods

### Experimental sites

**Figure 6:**
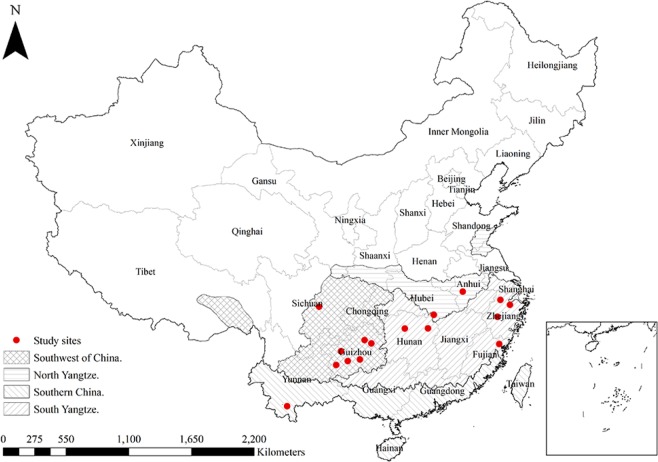
Distribution of experimental sites for model construction and validation (*n* = 21) for tea in four production regions of China. Points overlapped among the sites (two in Anhui, three in Hubei, three in Fujian) due to relatively small distance.

In China, regional tea cultivation varies based on climate, tea variety, and production history. Accordingly, tea-producing areas can be divided into four regions: the Southwest of China (SW), South Yangtze (SY), North Yangtze (NY), and Southern China (SC) (Fig. 6). Field experiments were conducted in Anhui, Hubei (NY), Yunnan (SC), Sichuan, Guizhou (SW), Hunan, Zhejiang, and Fujian (SY) provinces from 2016 to 2019. SW is the oldest tea-growing area in China, where several different varieties have originated. Annual precipitation in SW, which ranges from 900 to 1400 mm, is relatively ample^49^. The soil type in SW is mainly haplic acrisol. The soil types in SC and SY are mainly rhodic ferralsol and haplic acrisol. SY is dominated by a subtropical monsoon climate and is the most prolific of the tea-growing areas, accounting for 2/3 of the total tea production in China. We conducted three field experiments in Zhejiang province. Tea yield and output in Zhejiang province are highest in SY^30^. SC is the most arable area for tea, with rich hydrothermal resources. NY, the most northerly area, has mainly haplic luvisol soil and experiences large circadian temperature fluctuations, yielding a high-quality green tea^50^. A large number of soil types and climate conditions characterise these plots that represent a variety of soil chemical profiles (Supplementary Tables S4 and S5).

### Data sources

Data on different nutrient management practices including FP, OPT, and omission plots for N (OPT-N), P (OPT-P), and K (OPT-K) were gathered and collated. OPT fertilisation was as recommended by local tea experts (Table 1). The plot sizes of each experiment ranged from 30 to 54 m^2^ with a randomised block design. Local popular varieties of tea were planted in each field, and weeds, pests, and diseases were controlled. The tea plants were cultivated with optimised management practices including irrigation, trimming, and other established approaches. Tea plants were harvested as one bud with two young expanding leaves. The deactivation of tea enzymes was carried out in a microwave oven after harvest, and the tea was then oven-dried at 70 °C for 36 h. Then, the dry samples that were sieved through a 250 µm screen were digested with H_2_SO_4_-H_2_O_2_, and the Kjeldahl method, vanadium molybdate yellow colorimetry, and flame photometry were used to estimate N, P, and K concentrations, respectively^50^. We collected data on nutrient uptake and yield from tea field experiments in order to build the QUEFTS model.

**Table 1:** Rates of fertiliser application for optimum practice treatment (OPT).

Province	Case (n)	Fertiliser application rate (kg ha^−1^)		
		N	P_2_O_5_	K_2_O
Guizhou	6	330 (324–340)^b^	69 (57–133)	94 (68–139)
Sichuan	1	300	100	100
Yunnan	1	450	90	120
Anhui	2	360	120	120
Hunan	2	281 (273–288)	93 (90–96)	129 (102–156)
Zhejiang	3	359 (340–383)	92 (69–120)	104 (79–137)
Hubei	3	309 (263–339)	84 (68–100)	76 (72–80)
Fujian	3	338 (324–351)	145 (136–154)	115 (103–127)

^a^Data in parentheses indicates the range of fertiliser application.

### Model development

The QUEFTS model was first proposed by Janssen *et al*.^25^ to estimate maize yield in tropical areas without fertilisation. Smaling and Janssen^51^ improved the model to evaluate balanced N, P, and K uptake for a certain targeted yield after analysing substantial experimental data. In our study, we used the QUEFTS model to calculate the N, P, and K requirement for spring, summer, and autumn tea for a target yield. The core parameters of the QUEFTS model are the maximum accumulation (a) and maximum dilution (d) for tea plants. The values of (a) and (d) were calculated by excluding the upper and lower 2.5th, 5.0th, or 7.5th percentile of all measured IE data. Model-building followed four steps:

1. Identifying the relationship between soil chemical characteristics and soil nutrient supplements based on abundant data collected from field experiments. The main indexes of soil chemical characteristics are N, P, K, and organic matter.
2. Establishing a relationship between soil nutrient supply and plant nutrient uptake. The relationship between the potential supply of nutrients and actual absorption is based on the following considerations: First, the nutrients are paired for comparison. Therefore, the relationship between the actual uptake and the potential supply of N was calculated twice, resulting in two estimates of the actual uptake of each of the three nutrients. According to the law of the minimum, the lower of the two estimates is considered to be more realistic.
3. Determining the relationship between the actual absorption of N, P, and K and the range of yield based on data collected from fertilised and unfertilised crops.
4. Combining the production ranges into one production estimate. The process of combining the production ranges calculated in step 3 consists of two parts. The yield ranges are first combined in pairs (N and P, N and K, and P and K), and secondly, the average yield of nutrients is calculated. This average is an estimate of the final prediction of the actual yield^51^.

### Field validation

The field validation experiments performed in 2019 were conducted at the same sites as the field experiments performed to construct the QUEFTS model during 2016–2018 (*n* = 21; Fig. 6). The rates of fertiliser application were the same as those shown in Table 1. Urea was used as N fertiliser, with 30% applied one month before spring tea harvest, 20% applied between spring harvest and summer harvest, 20% applied between summer and autumn harvest, and 30% applied after autumn harvest. P and K fertiliser were applied as superphosphate and potassium sulfate, respectively, in a single treatment after autumn harvest. All fertilisers were applied in ditches between two rows of tea plants at a depth of 15 cm, then covered with soil^12^. The value of the RMSE and n-RMSE were used to evaluate the QUEFTS model and the deviation between the measured and simulated data. RMSE and n-RMSE were calculated using the following formula:

$$\begin{array}{ccc}{\rm{RMSE}} & = & \sqrt{\frac{{\sum }_{i=1}^{n}{(si-mi)}^{2}}{n}}\\ {\rm{Normalised}}\,{\rm{RMSE}} & = & \frac{{\rm{RMSE}}}{\overline{{\rm{m}}}}\end{array}$$

where *si* and *mi* represent simulated and measured values, respectively; *n* represents the number of measures; and represents the mean data measurement^52^.

### Statistical analysis

One-way analysis of variance (ANOVA) was performed using Statistical Package for Social Sciences (SPSS) version 20.0. Probability values (*P*) less than 0.05 were considered significant.

## Supplementary information

Supplementary information

## Data Availability

All data generated or analysed during this study are included in the manuscript file and its Supplementary Information files.
