# Supplementary material for: Temporal variation in nutrient requirements of tea (Camellia sinensis) in China based on QUEFTS analysis
Source: Sci Rep. 2020 Feb 4;10:1745. doi: 10.1038/s41598-020-57809-x (PMC7000836; doi:10.1038/s41598-020-57809-x)
Supplement: Supplementary file 1 — Supplementary information [file 41598_2020_57809_MOESM1_ESM.docx]

**Temporal variation in nutrient requirements of tea (*Camellia sinensis*) in China based on QUEFTS analysis**

Sheng Tang^1,2^, Yanling Liu^3^, Nan Zheng^1^, Yu Li^3^, Qingxu Ma^1^, Han Xiao^1^, Xuan Zhou^4^, Xinpeng Xu^5^, Taiming Jiang^6^, Ping He^5^ & Lianghuan Wu^1,2*^

**Fig. S1** Yield of spring, summer, autumn and all tea from 2016 to 2018. “all tea” represented the annual tea including spring, summer and autumn tea. Different letters represent significant differences at *P* < 0.05.


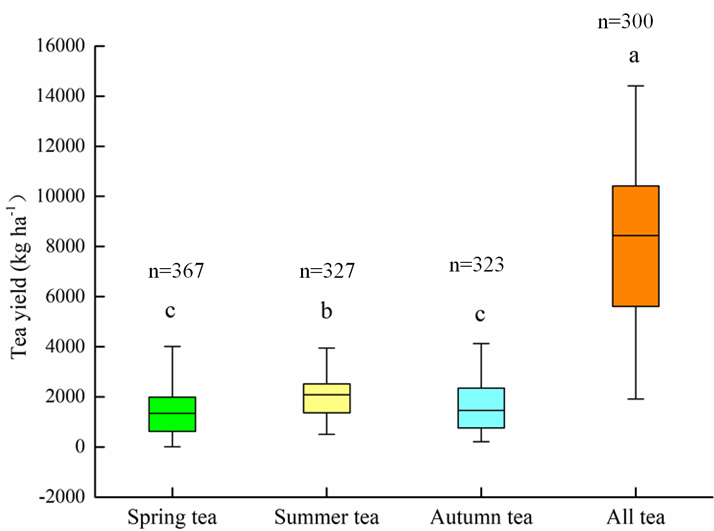


**Fig. S2** To produce 1000 kg tea, the nutrient requirements of spring, summer, autumn and all tea.


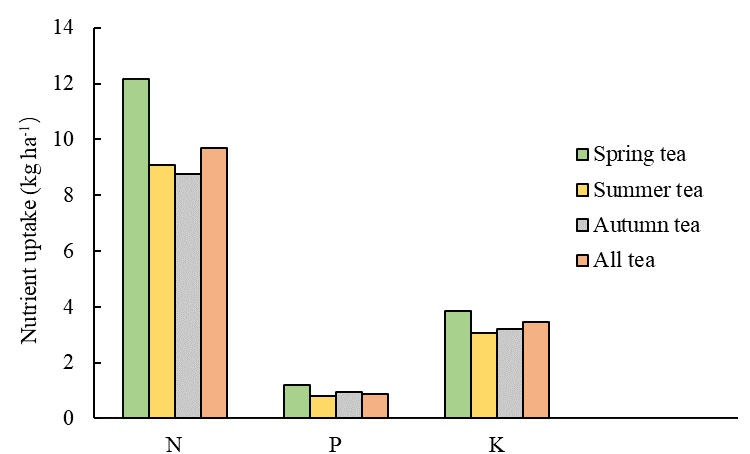


**Supplementary Table S1**. The nutrient uptake characteristics of tea in China.

| Data set | Parameter | Unit | *n*^a^ | Mean | SD^b^ | 25%*Q*^c^ | Median | 75%*Q* |
| --- | --- | --- | --- | --- | --- | --- | --- | --- |
| Spring tea | IE^d^-N | kg kg^-1^ | 367 | 75.7 | 12.8 | 67.2 | 74.4 | 82.3 |
|  | IE-P | kg kg^-1^ | 367 | 733.2 | 184.4 | 608.0 | 688.6 | 784.3 |
|  | IE-K | kg kg^-1^ | 367 | 239.0 | 53.5 | 196.4 | 230.8 | 282.0 |
|  | RIE^e^-N | kg t^-1^ | 367 | 13.5 | 2.1 | 12.2 | 13.4 | 14.9 |
|  | RIE-P | kg t^-1^ | 367 | 1.4 | 0.3 | 1.3 | 1.5 | 1.6 |
|  | RIE-K | kg t^-1^ | 367 | 4.4 | 0.9 | 3.6 | 4.3 | 5.1 |
| Summer tea | IE-N | kg kg^-1^ | 327 | 101.7 | 14.2 | 93.5 | 103.4 | 108.4 |
|  | IE-P | kg kg^-1^ | 327 | 1134.1 | 256.3 | 1000.0 | 1135.1 | 1250.0 |
|  | IE-K | kg kg^-1^ | 327 | 264.8 | 67.7 | 223.5 | 236.7 | 270.2 |
|  | RIE-N | kg t^-1^ | 327 | 10.0 | 1.3 | 9.2 | 9.7 | 10.7 |
|  | RIE-P | kg t^-1^ | 327 | 0.9 | 0.2 | 0.8 | 0.9 | 1.0 |
|  | RIE-K | kg t^-1^ | 327 | 4.0 | 0.7 | 3.7 | 4.2 | 4.5 |
| Autumn tea | IE-N | kg kg^-1^ | 323 | 106.0 | 18.4 | 93.1 | 104.7 | 119.2 |
|  | IE-P | kg kg^-1^ | 323 | 1063.9 | 230.2 | 888.9 | 1076.2 | 1256.1 |
|  | IE-K | kg kg^-1^ | 323 | 274.8 | 68.4 | 216.2 | 272.2 | 317.9 |
|  | RIE-N | kg t^-1^ | 323 | 9.7 | 1.8 | 8.4 | 9.6 | 10.8 |
|  | RIE-P | kg t^-1^ | 323 | 1.0 | 0.2 | 0.8 | 0.9 | 1.1 |
|  | RIE-K | kg t^-1^ | 323 | 3.9 | 0.9 | 3.2 | 3.7 | 4.6 |
| All tea | IE-N | kg kg^-1^ | 300 | 98.1 | 12.5 | 89.8 | 98.7 | 106.4 |
|  | IE-P | kg kg^-1^ | 300 | 1002.5 | 168.4 | 892.2 | 963.0 | 1066.9 |
|  | IE-K | kg kg^-1^ | 300 | 257.5 | 48.4 | 217.8 | 238.8 | 293.2 |
|  | RIE-N | kg t^-1^ | 300 | 10.4 | 1.4 | 9.4 | 10.1 | 11.1 |
|  | RIE-P | kg t^-1^ | 300 | 1.0 | 0.3 | 0.9 | 1.0 | 1.1 |
|  | RIE-K | kg t^-1^ | 300 | 4.0 | 0.7 | 3.4 | 4.2 | 4.6 |

^a^ *n*=number of observations.

^b^ SD=standard deviation.

^c^ *Q=*quartile.

^d^ IE=Internal efficiency (kg yield per kg nutrient uptake).

^e^ RIE=Reciprocal internal efﬁciency (nutrient uptake requirement per ton of yield).

**Supplementary Table S2**. The maximum accumulation (a) and dilution (d) values of three series (Set I, Set II and Set III) of spring, summer, autumn and all tea based on the datasets. Constant a and d were calculated by excluding the upper and lower 2.5 (Set I), 5.0 (Set II) and 7.5 (Set III) percentiles of all nutrient internal efficiency data of the combined data set (spring tea, summer tea, autumn tea and all tea).

| Data sets | Nutrients | Set I |  | Set II |  | Set III |  |
| --- | --- | --- | --- | --- | --- | --- | --- |
|  |  | a (2.5th) | d (97.5th) | a (5th) | d (95th) | a (7.5th) | d (92.5th) |
| Spring tea | N | 56 | 103 | 59 | 98 | 61 | 96 |
|  | P | 489 | 1193 | 522 | 1155 | 540 | 1127 |
|  | K | 161 | 356 | 170 | 342 | 173 | 326 |
| Summer tea | N | 78 | 131 | 80 | 118 | 82 | 114 |
|  | P | 727 | 1798 | 752 | 1535 | 765 | 1429 |
|  | K | 213 | 431 | 214 | 409 | 215 | 393 |
| Autumn tea | N | 75 | 141 | 76 | 136 | 80 | 131 |
|  | P | 642 | 1436 | 667 | 1384 | 703 | 1354 |
|  | K | 184 | 409 | 190 | 379 | 194 | 366 |
| All tea | N | 74 | 123 | 78 | 119 | 79 | 115 |
|  | P | 764 | 1404 | 802 | 1360 | 808 | 1306 |
|  | K | 205 | 344 | 207 | 333 | 209 | 328 |

**Supplementary Table S3**. The nutrient uptake characteristics and yield of field validation experiments in 2019.

| Data set | Parameter | Unit | *n*^a^ | Mean | SD^b^ | 25%*Q*^c^ | Median | 75%*Q* |
| --- | --- | --- | --- | --- | --- | --- | --- | --- |
| Spring tea | Yield | kg ha^-1^ | 73 | 1438.6 | 654.0 | 908.8 | 1443.2 | 1853.5 |
|  | IE^d^-N | kg kg^-1^ | 73 | 83.9 | 13.7 | 72.8 | 84.2 | 94.4 |
|  | IE-P | kg kg^-1^ | 73 | 849.7 | 225.1 | 676.4 | 813.0 | 990.3 |
|  | IE-K | kg kg^-1^ | 73 | 267.4 | 57.8 | 222.7 | 250.2 | 323.3 |
|  | RIE^e^-N | kg t^-1^ | 73 | 12.2 | 2.1 | 10.6 | 11.9 | 13.7 |
|  | RIE-P | kg t^-1^ | 73 | 1.3 | 0.3 | 1.0 | 1.2 | 1.5 |
|  | RIE-K | kg t^-1^ | 73 | 3.9 | 0.8 | 3.1 | 4.0 | 4.5 |
| Summer tea | Yield | kg ha^-1^ | 66 | 1040.1 | 1410.5 | 362.4 | 466.9 | 612.3 |
|  | IE-N | kg kg^-1^ | 66 | 106.2 | 20.2 | 92.1 | 101.2 | 117.8 |
|  | IE-P | kg kg^-1^ | 66 | 984.3 | 265.6 | 781.8 | 941.9 | 1099.4 |
|  | IE-K | kg kg^-1^ | 66 | 283.8 | 40.6 | 259.5 | 274.9 | 316.5 |
|  | RIE-N | kg t^-1^ | 66 | 9.7 | 1.8 | 8.5 | 9.9 | 10.9 |
|  | RIE-P | kg t^-1^ | 66 | 1.1 | 0.3 | 0.9 | 1.1 | 1.3 |
|  | RIE-K | kg t^-1^ | 66 | 3.6 | 0.5 | 3.1 | 3.6 | 3.9 |
| Autumn tea | Yield | kg ha^-1^ | 54 | 1146.3 | 1602.8 | 301.3 | 371.6 | 1751.9 |
|  | IE-N | kg kg^-1^ | 54 | 116.1 | 19.7 | 102.7 | 114.1 | 123.1 |
|  | IE-P | kg kg^-1^ | 54 | 1216.4 | 208.2 | 1056.2 | 1175.9 | 1317.7 |
|  | IE-K | kg kg^-1^ | 54 | 279.8 | 53.4 | 237.5 | 261.1 | 299.1 |
|  | RIE-N | kg t^-1^ | 54 | 8.8 | 1.4 | 8.1 | 8.8 | 9.7 |
|  | RIE-P | kg t^-1^ | 54 | 0.8 | 0.1 | 0.8 | 0.9 | 0.9 |
|  | RIE-K | kg t^-1^ | 54 | 3.7 | 0.6 | 3.3 | 3.8 | 4.2 |

**Supplementary Table S4.** Soil chemical properties of experimental sites for tea production in four regions of China

| Region | Province | Soil type | pH | Organic matter  (g kg^-1^) | Alkali-hydrolysable N  (mg kg^-1^) | Olsen P  (mg kg^-1^) | NH_4_OAc-K  (mg kg^-1^) |
| --- | --- | --- | --- | --- | --- | --- | --- |
| SW^a^ | Guizhou | Haplic Alisol | 3.7-6.0 | 17.4-68.4 | 63.7-353.5 | 0.5-166.2 | 87.0-1070.0 |
|  | Sichuan | Haplic Alisol | 3.9-4.9 | 12.4-28.7 | 103.6-105.9 | 12.1-13.8 | 145.0-159.0 |
| NY^b^ | Anhui | Eutric Regosol | 5.5 | 52.8 | 71.4 | 7.1 | 75.0 |
|  | Hubei | Haplic Alisol | 4.0-4.1 | 35.8-46.2 | 92.4-116.2 | 46.9-71.5 | 27.3-136.7 |
| SC^c^ | Yunnan | Haplic Acrisol | 4.1 | 35.1 | 222.5 | 17.5 | 251.0 |
| SY^d^ | Hunan | Haplic Acrisol | 4.4-4.7 | 23.7-57.1 | 44.1-124.0 | 26.4-202.7 | 20.3-86.0 |
|  | Fujian | Eurtic Vertisol | 4.4-4.7 | 27.1-30.9 | 102.6-124.7 | 27.6-86.9 | 141.9-157.4 |
|  | Zhejiang | Haplic Acrisol | 3.8-4.0 | 33.3-69.8 | 125.0-180.6 | 101.9-178.5 | 50.1-141.9 |
|  |  | Haplic Alisol |  |  |  |  |  |

^a^ SW = Southwest of China.

^b^ NY= North Yangtze.

^c^ SC = Southern China.

^d^ SY = South Yangtze.

**Supplementary Table S5.** Climate characteristics of experimental sites for tea production in four regions of China.

| Region | Province | Latitude (^。^N) | Longitude (^。^E) | Precipitation (mm) | *T*_min_^a^ | *T*_max_^b^ |
| --- | --- | --- | --- | --- | --- | --- |
| SW | Guizhou | 24.37-29.13 | 103.36-109.35 | 1100-1400 | 3 | 25 |
|  | Sichuan | 26.03-34.19 | 97.21-108.31 | 900-1200 | -2 | 20 |
| NY | Anhui | 29.41-34.38 | 114.54-119.37 | 700-1400 | -1 | 30 |
|  | Hubei | 29.01-33.06 | 108.21-116.07 | 800-1600 | −4 | 35 |
| SC | Yunnan | 24.23-26.22 | 102.10-103.40 | 600-2000 | 6 | 22 |
| SY | Hunan | 24.38-30.08 | 108.47-114.15 | 900-1700 | 4 | 35 |
|  | Fujian | 23.30-28.20 | 115.40-120.30 | 1400-2000 | 6 | 39 |
|  | Zhejiang | 27.02-31.11 | 118.01-123.10 | 980-2000 | -2 | 33 |

^a^ SW = Southwest of China.

^b^ NY= North Yangtze.

^c^ SC = Southern China.

^d^ SY = South Yangtze.

^a^ *T*_min_=minimum temperature.

^b^ *T*_max_=maximum temperature.
